# Rapunzel Syndrome Complicated by *Escherichia coli* Sepsis, Bowel Perforation, and Pancreatitis in an 11-year-old Malnourished Female

**DOI:** 10.1097/PG9.0000000000000171

**Published:** 2022-01-24

**Authors:** Rhea Daniel, Mary Arbuthnot, Srinivas Ramireddy, Supriya Nair

**Affiliations:** From the * Department of Pediatric Gastroenterology, Hepatology, and Nutrition, McGovern Medical School, University of Texas Health Science Center and Children’s Memorial Hermann Hospital, Houston, TX; † Department of Pediatric Surgery, McGovern Medical School, University of Texas Health Science Center and Children’s Memorial Hermann Hospital, Houston, TX; ‡ Department of Gastroenterology, McGovern Medical School, University of Texas Health Science Center, Houston, TX.

**Keywords:** Rapunzel syndrome, trichobezoar, sepsis, pancreatitis

## Abstract

The most common presenting symptoms of Rapunzel syndrome include abdominal pain (37%), nausea and vomiting (33.3%), obstruction (25.9%), and peritonitis (18.3%). Less commonly, patients may present with weight loss (7.4%) or intussusception (7.4%). Exceedingly rare complications of Rapunzel syndrome include gastric ulceration, obstructive jaundice, and acute pancreatitis as well as other malabsorptive-related complications including protein-losing enteropathy, iron deficiency, and megaloblastic anemia. This report details the case of an 11-year-old female with Rapunzel syndrome complicated by sepsis, a rare complication reported in only 2% of patients.

Rapunzel syndrome, first described in 1968 by Vaughn et al, is a rare form of a trichobezoar with bezoar extension into the small bowel.^1^ The most common presenting symptoms of Rapunzel syndrome include abdominal pain (37%), nausea and vomiting (33.3%), obstruction (25.9%), and peritonitis (18.3%).^2^ Less commonly, patients may present with weight loss (7.4%) or intussusception (7.4%).^2^ Exceedingly rare complications of Rapunzel syndrome include gastric ulceration, obstructive jaundice, and acute pancreatitis as well as other malabsorptive related complications including protein-losing enteropathy, iron deficiency, and megaloblastic anemia.^3^ A 2016 structured PubMed search reviewing Rapunzel syndrome with complications reported sepsis occurring in only 2% of patients.^4^ We reviewed the literature since 2016 and found only 1 additional report of sepsis occurring as a complication of Rapunzel syndrome.^5^ Here, we present a case of Rapunzel syndrome in an 11-year-old female with the infrequently reported complication of *Escherichia coli* sepsis as well as malnutrition, pancreatitis, cholangitis, and bowel perforation.

## CASE REPORT

Our patient is an 11-year-old female with a 5-year history of poor weight gain, presenting with a 3-week history of constant nonradiating epigastric abdominal pain, nausea, vomiting, and decreased appetite. She was initially evaluated by her primary care provider at symptom onset and was prescribed a proton pump inhibitor and dicyclomine for pain, which did not provide relief. Upon arrival to the emergency department, physical exam was notable for malnutrition, with a weight z-score for age –2.15 and BMI < first percentile (CDC 2–20 years, girls); dehydration, with heart rate of 109 beats per minutes; and mild epigastric abdominal pain. Initial labs were notable for an albumin of 2.9 g/dL, ALT 151 µ/L, AST 100 µ/L, total bilirubin 0.4 mg/dL, direct bilirubin 0.1 mg/dL, lipase 1030 µ/L, triglycerides 65 mg/dL, WBC 11.7 K/mm^3^ (81.9% neutrophils, 10.5% lymphocytes), Hg 14.4 g/dL, and MCV 83.3 fL. A right upper quadrant ultrasound was obtained revealing a normal gallbladder with no gallstones and a common bile duct measuring 11 mm. Pancreas was not visualized on ultrasound.

**FIGURE 1.**
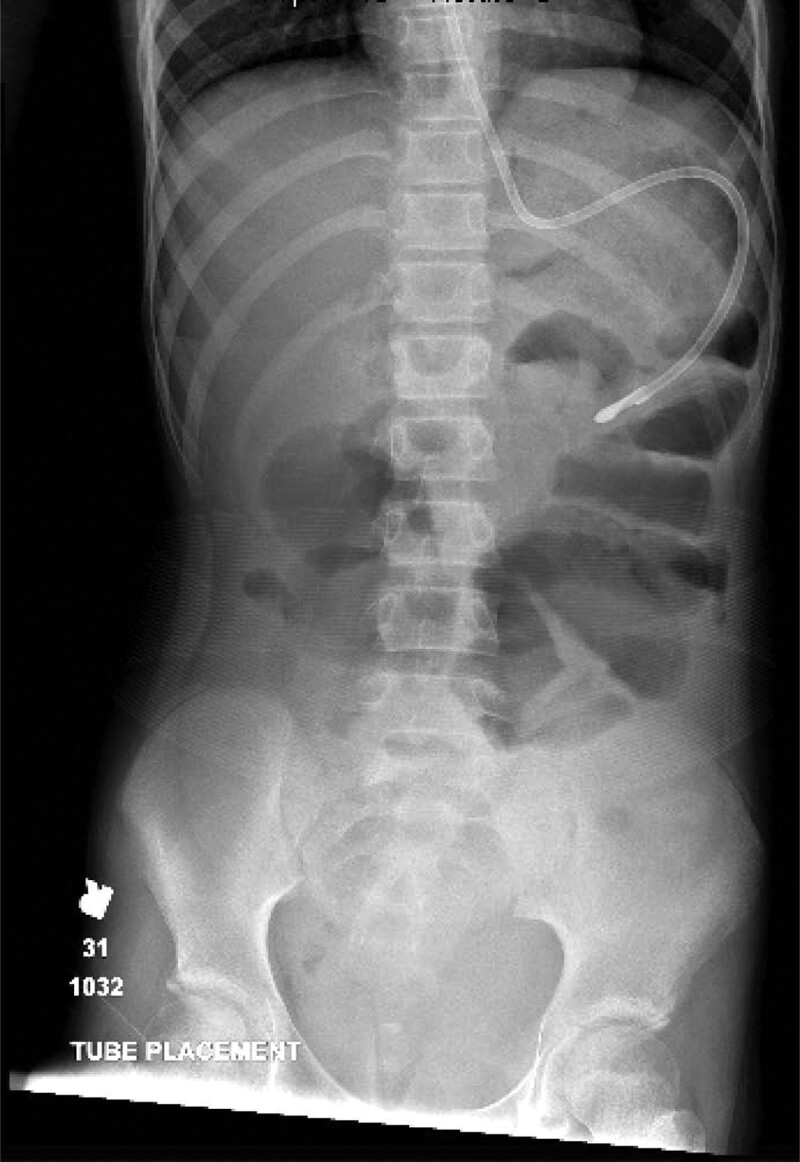
NGT taking an unusual course along the periphery of the stomach. NGT indicates nasogastric tube.

She was admitted with intravenous fluids as well as ketorolac and morphine for pain control. On hospital day 2, she became febrile to 102.1°F, measured orally. Blood and urine cultures were obtained before initiating piperacillin-tazobactam. Blood cultures returned positive for *E. coli* within 6 hours of collection. A nasogastric tube was placed for gastric decompression due to nausea and vomiting. An abdominal x-ray was obtained to confirm NGT position and revealed the NGT was appropriately placed; however, the tube was noted to take an unusual course along the periphery of the stomach (Fig. 1). Repeat labs revealed an ALT of 419 µ/L, AST 720 µ/L, total bilirubin 1 mg/dL, and direct bilirubin 0.6 mg/dL. A magnetic resonance cholangiopancreatogram was obtained and revealed no intraductal stones. A nonlayering low signal gastric substance was identified along with fluid-filled loops of small bowel. The pancreas appeared normal. Liver function tests remained elevated and bilirubin continued to increase (total bilirubin 2.2 mg/dL, and direct bilirubin 1.6 mg/dL). She underwent upper endoscopy on hospital day 3, which revealed a large trichobezoar occupying most of the stomach and extending past the pylorus into the third portion of the duodenum. Visualization of the ampulla was obscured due to proximity of the trichobezoar, but it was noted to be prominent and erythematous.

**FIGURE 2.**
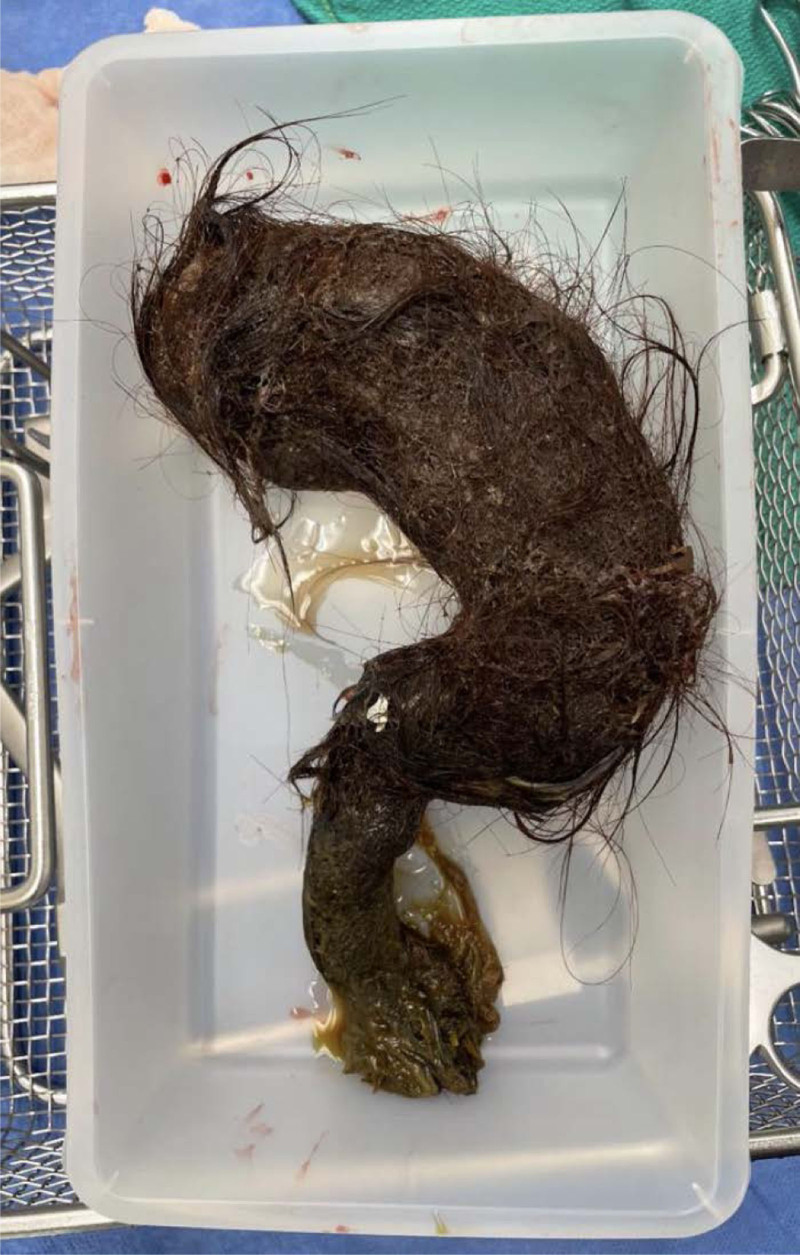
Large gastric bezoar containing hair, lice, nail clippings, and plant-like material extending from the stomach to the terminal ileum with several rope-like turns resulting in kinking of the small bowel.

**FIGURE 3.**
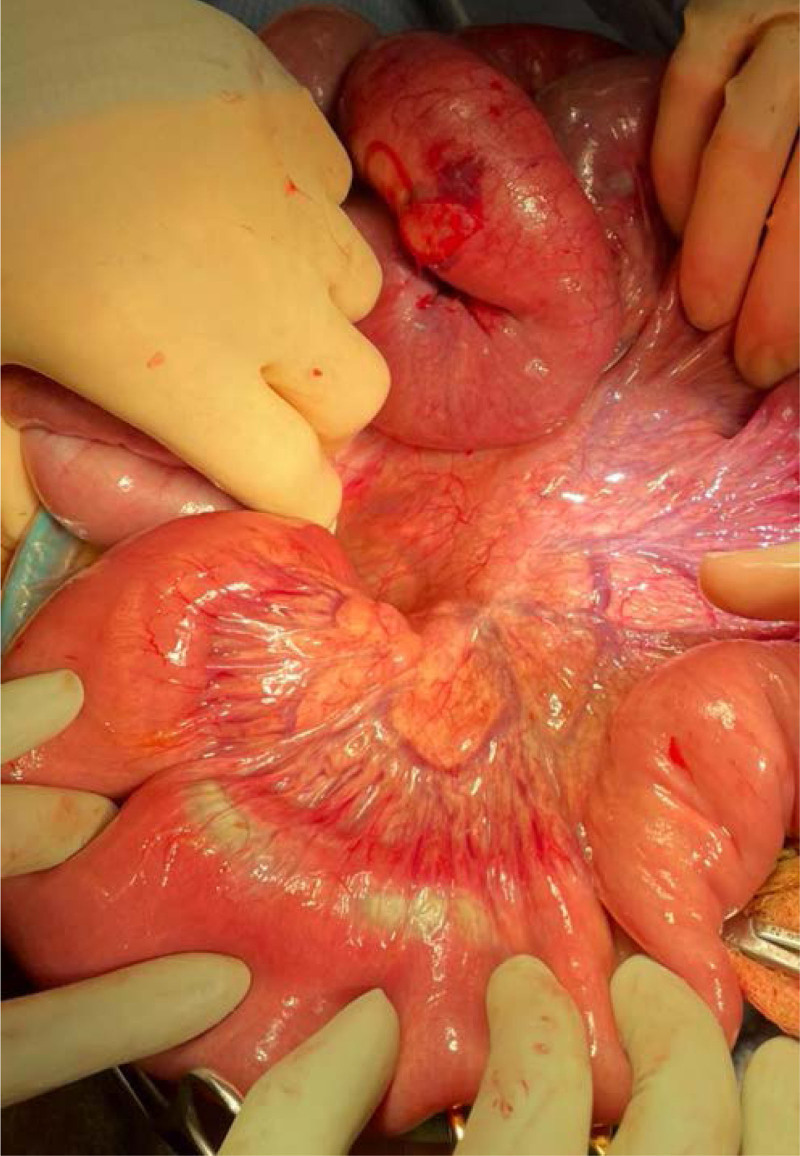
Several areas of full thickness necrosis noted along the mesenteric border of the small bowel.

The patient underwent emergent laparotomy, which revealed a large gastric bezoar containing hair, lice, nail clippings, and plant-like material extending from the stomach to the terminal ileum with several rope-like turns resulting in kinking of the small bowel (Fig. 2). Several areas of full thickness necrosis were noted along the mesenteric border of the small bowel (Fig. 3). The bezoar was extracted through a gastrostomy and 2 small bowel enterotomies. A temporary abdominal closure was placed with a second look performed 48 hours later. Fluconazole was started, and the patient was treated with topical permethrin for lice. She remained intubated and developed disseminated intravascular coagulation and hypotension requiring vasopressor support. Second look revealed multiple patches of partial thickness necrosis along the mesenteric border and a full thickness area of necrosis, which had perforated. The bowel was repaired in single-layer fashion. A temporary abdominal closure was placed once again due to bowel edema. Abdominal closure was performed 72 hours later, and the patient was able to be successfully extubated. She completed a 14-day course of piperacillin/tazobactam and a 7-day course of fluconazole. Further workup revealed an iron level <10 µg/dL and total iron binding capacity of 144 µg/dL.

Psychiatry evaluated the patient before discharge, and she endorsed trichotillomania and trichophagia. Her parents and siblings denied ever witnessing her pulling or eating her hair or consuming any nonedible material. She had no known history of compulsive behaviors such as nail biting. On physical exam, she had no visible hair thinning or areas of alopecia. Further dietary history revealed the patient had a fair appetite at home eating 3 meals a day and snacks; however, she was a very picky eater as long as parents could recall and ate the same meals every day, typically pasta and chips, and refused to try new foods. Parents reported being concerned about her protein intake. When protein was encouraged and provided at meals, she would start gagging. Parents denied ever being told by her pediatrician that she was underweight. Cognitive behavioral therapy was recommended, and she was discharged to an inpatient nutritional and physical rehabilitation program.

## DISCUSSION

Trichotillomania is classified as an obsessive-compulsive disorder with onset usually between the ages of 10–13 years old with a female predominance in late adolescence.^6^ In the pediatric population, lifetime prevalence is around 1%–3%, and only a subset of patients will have trichophagia leading to trichobezoar.^6^ Rapunzel syndrome is rare form of trichobezoar with extension into the small bowel. It most commonly presents with abdominal pain and nausea/vomiting.^2^ Symptoms of severe malnutrition, acute pancreatitis, and bowel perforation are extremely rare. A literature search in 2016 by Ullah et al revealed only 2% of patients with Rapunzel syndrome had the complication of sepsis.^4^ The reports of sepsis describe a 5-year-old neglected female with a fatal outcome and another 5-year-old female with few details provided about the case.^4^ Our literature review revealed only 1 additional case of Rapunzel syndrome complicated by sepsis reported since then.^5^ This case occurred in a 17-year-old female with a 5-month history of abdominal pain acutely worsening 2 weeks before presentation.^5^ Since bowel perforation, although still a rare complication, is reported more frequently than sepsis, it maybe that subacute symptoms of abdominal pain and delayed presentation contribute to the development of sepsis. Our patient reported a 3-week history of abdominal pain, and the aforementioned cases described ongoing pain for 5 months and an unspecified, but presumably prolonged, amount of time in the case of neglect. To our knowledge, this is the first case reported in the literature of a patient with Rapunzel syndrome having concurrent *E. coli* sepsis, bowel perforation, and acute pancreatitis.^3^ Acute pancreatitis is thought to occur due to irritation and obstruction of the ampulla of Vater by mass effect of the bezoar tail as evidenced by a prominent and erythematous ampulla visualized on endoscopy. Trichobezoars may go unrecognized until they are large enough to cause gastrointestinal symptoms, as there may not be obvious signs of trichotillomania on exam, and the behavior often occurs in private. Cognitive behavioral therapy is the cornerstone of treatment, and a thorough psychiatric evaluation is warranted given the high incidence of other comorbid conditions such as mood, anxiety, eating, and substance use disorders.^6^
